# Molecular characterization and comparison of *bla*_NDM-1_-carrying and *bla*_NDM-5_-harboring IncX3-type plasmids in carbapenem-resistant *Klebsiella pneumoniae*

**DOI:** 10.1128/spectrum.01028-23

**Published:** 2023-08-25

**Authors:** Yunxing Yang, Haiyang Liu, Lingxia Chen, Minjie Mao, Xiaofan Zhang, Longjie Zhou, Darong Duan, Xi Li, Hua Zhou

**Affiliations:** 1 Department of Clinical Laboratory, Affiliated Hangzhou First People’s Hospital, Zhejiang School of Medicine, Hangzhou, China; 2 Laboratory Medicine Center, Department of Clinical Laboratory, Zhejiang Provincial People’s Hospital (Affiliated People’s Hospital, Hangzhou Medical College), Hangzhou, Zhejiang, China; 3 Department of Clinical Laboratory, The First Hospital of Jiaxing, Affiliated Hospital of Jiaxing University, Jiaxing, Zhejiang, China; 4 Department of Laboratory Medicine, Huangyan Hospital of Wenzhou Medical University, Taizhou First People’ s Hospital, Taizhou, Zhejiang, China; 5 Department of Respiratory and Critical Care Medicine, The First Affiliated Hospital, Zhejiang University School of Medicine, Hangzhou, Zhejiang, China; Memorial Sloan Kettering Cancer Center, New York, New York, USA

**Keywords:** carbapenemases, NDM-1, NDM-5, IncX3, fitness

## Abstract

Carbapenem-resistant *Klebsiella pneumoniae* (CRKP), which harbors the *bla*_NDM_ plasmid, has been reported extensively and is considered a global threat clinically. However, characterization and comparisons of *bla*_NDM-1_-carrying and *bla*_NDM-5_-harboring IncX3-type plasmids in CRKP are lacking. Here, we systematically compared the differences in the characteristics, genetic backgrounds, transferability, and fitness costs between *bla*_NDM-1_-carrying and *bla*_NDM-5_-carrying plasmids in *K. pneumoniae* isolates. Fifteen NDM-producing CRKP isolates were recovered from 1376 CRKP isolates between 2019 and 2021, of which 4 were positive for *bla*_NDM-1_ and 11 were positive for *bla*_NDM-5_. All strains were highly resistant to carbapenem but remained susceptible to tigecycline and colistin. Core-genome-based phylogenetic analyses revealed that these strains were not clonally related. Whole-genome sequencing showed that *bla*_NDM-1_ and *bla*_NDM-5_ were located on ~54 kb and ~46 kb IncX3-type plasmids, respectively. The backbone, genetic context, and fitness cost of the *bla*_NDM-1_-bearing plasmid were highly similar to those of the *bla*_NDM-5_-carrying plasmid, but the transferability of the *bla*_NDM-1_-positive plasmid was greater than that of the *bla*_NDM-5_-positive plasmid. In conclusion, the transmission of *bla*_NDM-1_ or *bla*_NDM-5_ is mainly disseminated by plasmids rather than clonal spread. The high transfer frequency of the IncX3 plasmid facilitates the prevalence and dissemination of NDM-KP among *Enterobacteriaceae*.

**IMPORTANCE:**

The emergence of NDM-producing *Klebsiella pneumoniae* is a severe challenge to public health. The widespread presence of *bla*_NDM-1_ and *bla*_NDM-5_ in *Enterobacteriaceae* has aroused broad concern. In this study, we performed molecular characterization of *bla*_NDM-1_-carrying and *bla*_NDM-5_-harboring IncX3-type plasmids in carbapenem-resistant *Klebsiella pneumoniae* (CRKP) and compared their phenotypes between strains with different *bla*_NDM_ subtype. Our findings highlight the importance of IncX3-type plasmids in the transfer of the *bla*_NDM-1_ and *bla*_NDM-5_ genes and demonstrate that the *bla*_NDM-1_ plasmid possesses higher transfer ability. These data will provide important insights into carbapenem resistance gene transfer via plasmids and their further spread in clinical settings.

## INTRODUCTION

Carbapenem-resistant *Klebsiella pneumoniae* (CRKP) is a serious global health threat (1). New Delhi metallo-β-lactamase (NDM) is an important carbapenemase with the ability to hydrolyze almost all β-lactams, even carbapenems (2). NDM-1 was first identified in *K. pneumoniae* in 2008 (3), and an increasing number of variants have since been reported. NDM-5 differs from NDM-1 by only two amino acid substitutions and confers elevated carbapenem and expanded-spectrum cephalosporin resistance (4).

The dissemination and prevalence of NDM-1 and NDM-5 in *K. pneumoniae* and other *Enterobacteriaceae* are largely due to the plasmid-mediated transfer of *bla*_NDM_. The *bla*_NDM_ gene was reported to be located on different incompatibility typing plasmids, such as IncX3, IncFII, IncN, and IncF (5). The horizontal spread of *bla*_NDM_ is mediated by plasmids in many *Enterobacteriaceae* species. *Escherichia coli* and *K. pneumoniae* are the major hosts of *bla*_NDM_, followed by *Citrobacter freundii* and *Salmonella enterica* (2). Current research has raised great concern for when hypervirulent *K. pneumoniae* strains acquire a *bla*_NDM_-carrying plasmid. The wide spread of conjugative *bla*_NDM_-carrying plasmids has contributed to the emergence and prevalence of CR-hvKp strains (6).

In this study, we compared *bla*_NDM-1_-positive plasmid and *bla*_NDM-5_-bearing plasmid in CRKP isolates to elucidate the dissemination mechanism and confirm the horizontal gene transfer of *bla*_NDM_ among *Enterobacteriaceae*.

## RESULTS

### Bacterial strains and antimicrobial susceptibility

Among 15 NDM-KP isolates collected from two different hospitals (A and B), 4 *bla*_NDM-1_-producing *K. pneumoniae* and 11 *bla*_NDM-5_-positive *K. pneumoniae* were identified (Table 1). In this study, NDM-KP isolates were recovered from adult patients with age ranging from 42 to 88 and were mainly collected from sputum (40%, 6/15) and urine (26.67%, 4/15). The antimicrobial susceptibility testing results showed that all strains were resistant to carbapenem (MIC >128 mg/L), ceftazidime (MIC >128 mg/L), cefepime (MIC >128 mg/L), amoxicillin-clavulanic acid (MIC >128 mg/L), and ceftazidime-avibactam (MIC >128/4 mg/L), but most were susceptible to tigecycline (MIC 0.25–4 mg/L) and colistin (MIC 0.25–2 mg/L) (Table 2). In addition, 12 isolates were susceptible to amikacin (MIC 0.5–8 mg/L), and 3 isolates were resistant (MIC >128 mg/L). Concerning ciprofloxacin, three isolates were intermediate, and others were resistant (MIC 4- > 128 mg/L).

**TABLE 1:** Clinical information of the 15 NDM-KP isolates in the study

Strain	Hospital	Age	Sex	Specimen	Diagnosis
ZRY2400	A	68	Male	Blood	Bloodstream infection, esophageal tumor
NB0030	B	56	Female	Sputum	Pneumonia
NB0011	B	63	Male	Sputum	Pneumonia, gastric cancer
NB0010	B	58	Female	Blood	Bloodstream infection, acute myelogenous leukemia
NB1827	B	70	Male	Ascites	Bile duct infection
ZRY3974	A	51	Male	Urine	Pneumonia, hemiplegia
ZRY3951	A	72	Male	Sputum	Bronchiectasis
ZRY4277	A	81	Female	Sputum	Pneumonia, cerebral infarction
ZRY4810	A	51	Female	Sputum	Pneumonia, brainstem hemorrhage
ZRY5867	A	89	Male	Sputum	Pneumonia, Alzheimer’s disease
ZRY1178	A	58	Female	Urine	Urinary tract infection, hemiplegia
ZRY9668	A	60	Male	Blood	Bloodstream infection, respiratory failure
ZRY2312	A	88	Female	Urine	Urinary tract infection, hypertension
ZRY4942	A	76	Male	Catheter	Hemiplegia
NB0045	B	42	Male	Urine	Urinary tract infection, cerebral hemorrhage

**TABLE 2:** Antimicrobial susceptibility of the 15 NDM-KP isolates in the study

Strain	NDM type	ST	MIC (mg/L)^*a*^										
			FEP	CAZ	AMC	AK	CIP	IPM	MEM	ETP	TGC	CST	CZA
ZRY2400	NDM-1	152	>128	>128	>128	>128	8	>128	>128	>128	0.25	0.25	>128/4
NB0030	NDM-1	45	>128	>128	>128	8	16	>128	>128	>128	2	0.5	>128/4
NB0011	NDM-1	3303	>128	>128	>128	2	2	>128	>128	>128	0.25	0.25	>128/4
NB0010	NDM-1	12	>128	>128	>128	8	4	>128	>128	>128	1	0.5	>128/4
NB1827	NDM-5	1	>128	>128	>128	0.5	>128	>128	>128	>128	1	0.5	>128/4
ZRY3974	NDM-5	17	>128	>128	>128	1	>128	>128	>128	>128	0.5	1	>128/4
ZRY3951	NDM-5	17	>128	>128	>128	2	32	>128	>128	>128	0.5	2	>128/4
ZRY4277	NDM-5	17	>128	>128	>128	2	32	>128	>128	>128	0.5	0.5	>128/4
ZRY4810	NDM-5	17	>128	>128	>128	2	32	>128	>128	>128	1	0.5	>128/4
ZRY5867	NDM-5	1326	>128	>128	>128	2	4	>128	>128	>128	0.5	0.25	>128/4
ZRY1178	NDM-5	37	>128	>128	>128	0.5	64	>128	>128	>128	4	0.5	>128/4
ZRY9668	NDM-5	17	>128	>128	>128	8	2	>128	>128	>128	0.25	1	>128/4
ZRY2312	NDM-5	17	>128	>128	>128	4	2	>128	>128	>128	0.5	0.25	>128/4
ZRY4942	NDM-5	15	>128	>128	>128	>128	>128	>128	>128	>128	1	0.25	>128/4
NB0045	NDM-5	5837	>128	>128	>128	>128	>128	>128	>128	>128	0.25	0.25	>128/4
ZRY2400J53	–^*b*^	–	64	>128	>128	16	≤0.125	64	64	>128	≤0.0625	0.25	>128/4
NB1827J53	–	–	>128	>128	>128	8	≤0.125	>128	>128	>128	0.0625	0.25	>128/4
J53	–	–	≤0.125	≤0.125	2	1	≤0.125	0.5	0.25	≤0.125	≤0.0625	0.25	0.125

^*a*^ FEP, cefepime; CAZ, ceftazidime; AMC, amoxicillin-clavulanic acid; AK, amikacin; CIP, ciprofloxacin; IPM, imipenem; MEM, meropenem; ETP, ertapenem; TGC, tigecycline; CST, colistin; CZA, ceftazidime/avibactam.

^*b*^ –, not applicable.

**Fig 1:**
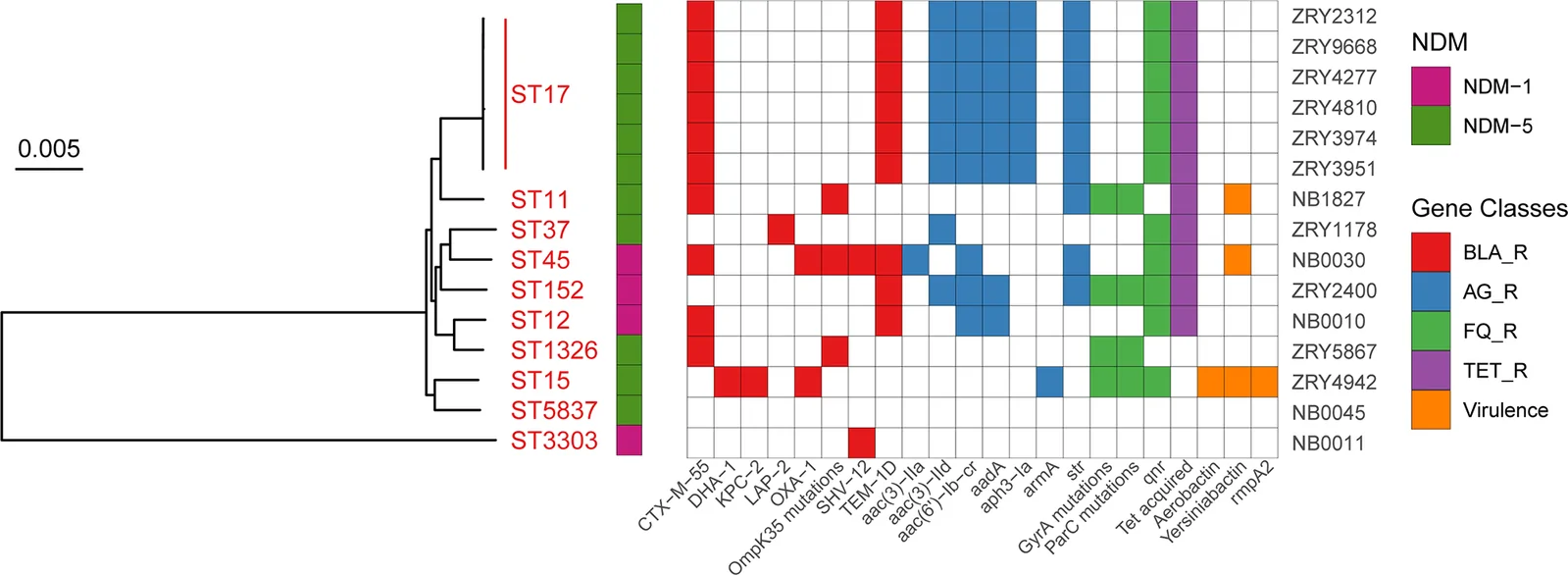
Core genome phylogenetic tree and gene heatmap of 15 NDM-KP strains. Red dots represent *K. pneumoniae* with *bla*_NDM-1_, and green dots represent *K. pneumoniae* with *bla*_NDM-5_. The gene heatmap contains resistance genes and virulence genes.

To uncover the resistance genes and virulence genes in the 15 isolates, we selected all strains for genome sequencing using the Illumina HiSeq sequencing platform. The distribution of resistance genes and virulence genes in the 15 isolates is listed in Fig. 1. Three isolates were positive for irp/ybtAEPQSTUX (encoding yersiniabactin), and only one isolate, ZRY4942, harbored iucABCD-iutA (encoding aerobactin) and rmpA2 (encoding mucoid-phenotype regulators); others were negative for these virulence genes.

Ten of the 15 isolates carried the extended-spectrum beta-lactamase (ESBL) gene *bla*_CTX-M-55_, and two isolates carried *bla*_SHV-12_. In addition, majority of the isolates harbored *bla*_TEM-1D_. Of note, isolate ZRY4942 carried *bla*_KPC-2_ and *bla*_NDM-5_, but none co-carried another *bla*_OXA-48_-like carbapenemase gene.

### Genetic relatedness

To investigate the phylogenetic relationship and genetic relatedness of NDM-KP isolates, multilocus sequence typing (MLST) and core genome phylogenetic tree analyses were carried out (Fig. 1). Six NDM-5-KP isolates belonged to the same sequence type, ST 17, and they were the most closely related to each other in the core genome phylogenetic alignment cluster. Others belonged to nine different sequence types (STs) (Table 2). In accordance with the MLST results, these strains displayed various clusters in the core genome phylogenetic tree.

### Characterization and location of the *bla*_NDM_ gene

**Fig 2:**
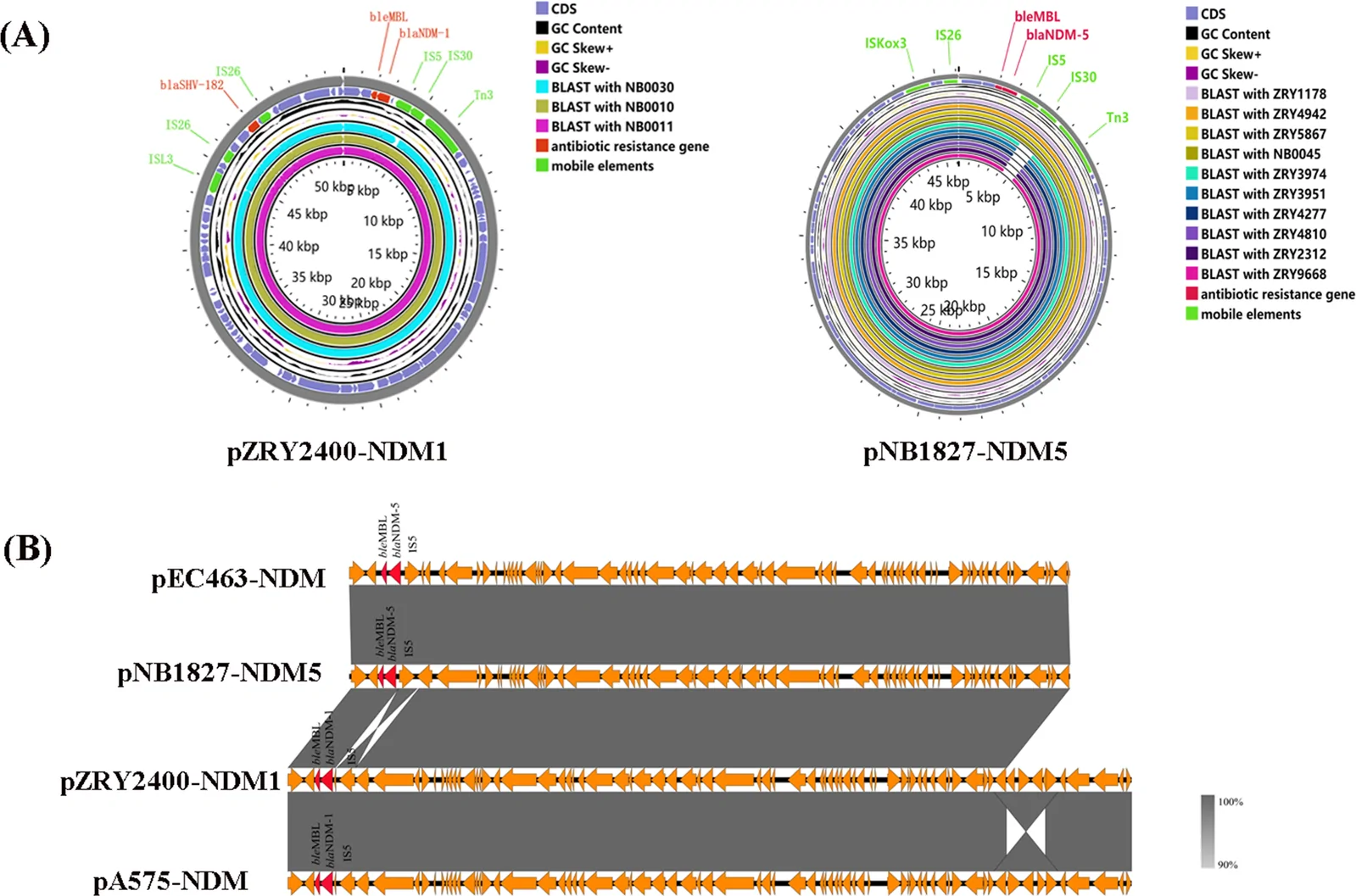
Genomic and molecular analyses of the *bla*_NDM-1_-positive plasmid pZRY2400-NDM1 and *bla*_NDM-5_-positive plasmid pNB1827-NDM5. (**A**) Schematic map of the pf plasmids pZRY2400-NDM1 and pNB1827-NDM5. (**B**) Comparative analysis of *bla*_NDM_-carrying IncX3 plasmids.

To determine the location of the *bla*_NDM_ gene, we selected two isolates, ZRY2400 and NB1827, as representatives for sequencing by Nanopore technology. In isolate ZRY2400, *bla*_NDM-1_ was located on a 54,035-bp IncX3 plasmid, pZRY2400-NDM1, with a GC content of 49.0% that was predicted to harbor 74 open reading frames (ORFs). Illumina sequence reads from other NDM-1-KP strains were mapped to pZRY2400-NDM1 with high identity and coverage, supporting the presence of a similar IncX3 plasmid in the *bla*_NDM-1_-carrying isolates (Fig. 2A).

In isolate NB1827, *bla*_NDM-5_ was located on a 46,161-bp plasmid, pNB1827-NDM5, with a 46.7% GC content that belonged to the IncX3 group and did not harbor any resistance genes other than *bla*_NDM-5_. The complete sequence of pNB1827-NDM5 was predicted to harbor 66 ORFs with a 46.7% GC content. BLAST analysis showed that the plasmids with *bla*_NDM-5_ in other strains were highly homologous to pNB1827-NDM5 (Fig. 2A).

### Plasmid sequence and comparative analysis

BLASTN search of GenBank showed that pZRY2400-NDM1 matched well with the *bla*_NDM-1_-carrying plasmid pA575-NDM (100% identity, 100% coverage) (GenBank accession no. NZ_MH917283.1), which is a typical *bla*_NDM-1_-positive plasmid from KP. The other plasmid pNB1827-NDM5 in our study was nearly identical to plasmid pEC463-NDM (GenBank accession no. MG545911) (99.95% identity and 100% coverage). The backbones of the *bla*_NDM-5_-positive plasmid pNB1827-NDM5 were highly similar to most of those in the *bla*_NDM-1_-carrying plasmid pZRY2400-NDM1, which indicated that integration or detachment events may have occurred during plasmid evolution.

In the ZRY2400 strain, the genetic environment of the *bla*_NDM-1_ gene (△IS*Aba125*-IS*5- bla*_NDM-1_-*ble*_MBL_-trpF-IS*26*) was identical to that of pA575-NDM. In the NB1827 strain, the *bla*_NDM-5_ gene was flanked in the upstream region by △IS*Aba125*-IS*5* and downstream by *ble*_MBL_-trpF-IS*26*-△umuD-IS*Kox3*, and this genetic context is the same as that of plasmid pEC463-NDM (Fig. 2B). Structural differences in the genetic elements surrounding *bla*_NDM-1_ and *bla*_NDM-5_ were observed only in the orientation of IS*5*.

### Transmissibility and fitness cost of plasmids bearing *bla*_NDM-1_ and *bla*_NDM-5_

To explore the transferability of plasmids with *bla*_NDM-1_ and *bla*_NDM-5_, a conjugation experiment was performed. The pZRY2400-NDM1 plasmid was transferred to *E. coli* J53 by conjugation at a frequency of 8.7 × 10^−2^ (transconjugant/recipient). The conjugation frequency of pNB1827-NDM5 (recipient strain, *E. coli* J53) was 4.3 × 10^−4^. The transconjugant displayed a similar antibiotic resistance phenotype to strains ZRY2400 and NB1827, whereas the MICs of imipenem, meropenem, and cefepime of the transconjugant NB1827J53 with the *bla*_NDM-5_-carrying plasmid were all >128 mg/L, which were higher than that of the transconjugant ZRY2400J53 with the *bla*_NDM-1_-carrying plasmid (64 mg/L).

**Fig 3:**
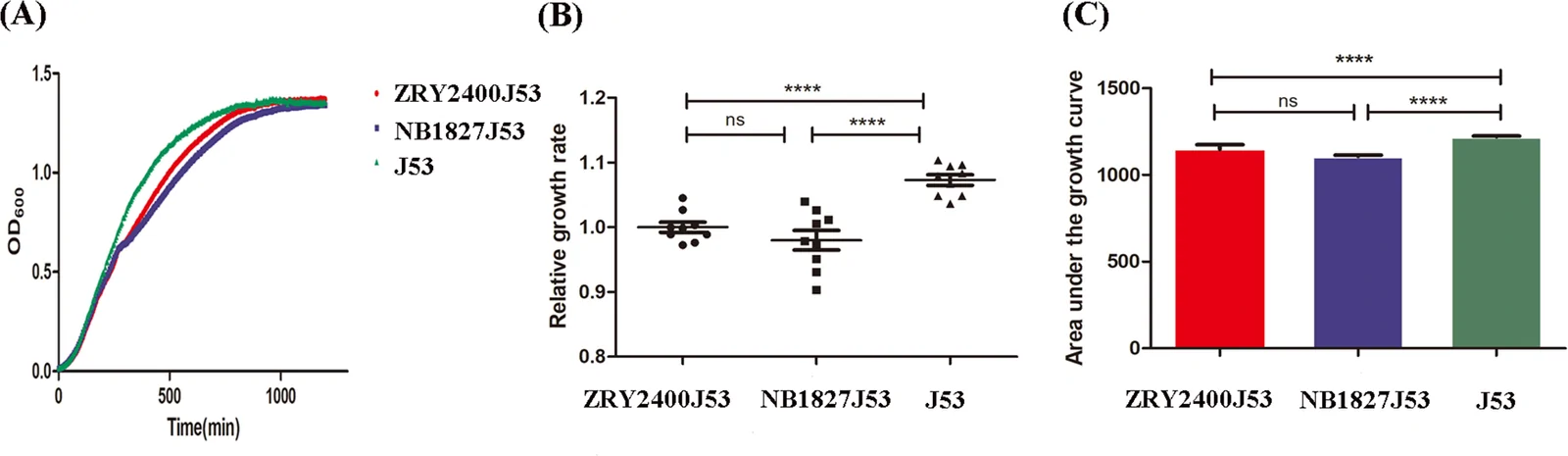
(**A**) Bacterial growth curve of strains ZRY2400J53, NB1827J53, and J53. OD600, optical density at 600 nm. (**B**) Relative growth rates of strains ZRY2400J53, NB1827J53, and J53. (**C**) Area under the growth curve of strains ZRY2400J53, NB1827J53, and J53. ****, *P* < 0.0001.

To compare the fitness of the *bla*_NDM_-harboring plasmid, growth kinetics assays were performed with strains with and without the plasmids pZRY2400-NDM1 or pNB1827-NDM5. Notably, a significant decrease was observed in the growth rate and area under the growth curve of the transconjugants ZRY2400J53 and NB1827J53 compared with the recipient *E. coli* J53 strain (*P* < 0.001) (Fig. 3). Results showed impaired growth as a result of the acquisition of plasmid pZRY2400-NDM1 or pNB1827-NDM5. However, no differences in the growth dynamics between the two transconjugants ZRY2400J53 and NB1827J53 were observed, indicating that the fitness burden of the *bla*_NDM-1_-positive plasmid and *bla*_NDM-5_-positive plasmid to the host was similar.

## DISCUSSION

Carbapenem resistance in *K. pneumoniae* is an urgent clinical problem that needs to be solved. Carbapenemase NDM remains a severe challenge and concern of many studies (2, 5). To date, carbapenemases NDM-1 and NDM-5 have been described mostly in *Enterobacteriaceae* isolates. However, few studies have systematically compared the differences between *bla*_NDM-1_-carrying and *bla*_NDM-5_-carrying plasmids. Hence, we compared the differences in the characteristics, genetic background, transferability, and fitness cost between the *bla*_NDM-1_-carrying plasmid and *bla*_NDM-5_-carrying plasmid in *K. pneumoniae* isolates obtained in the local region. Our results revealed that the IncX3-type plasmid played a vital role in the transport of *bla*_NDM-1_ and *bla*_NDM-5_ in *K. pneumoniae*, and the backbone, genetic context, and fitness cost of *bla*_NDM-1_ were highly similar to those of the *bla*_NDM-5_-carrying plasmid; however, the transferability of the *bla*_NDM-1_-positive plasmid was greater than that of the *bla*_NDM-5_-positive plasmid. Taken together, our findings suggest that the emerging threats of plasmid-mediated transfer and spread of *bla*_NDM_ in *K. pneumoniae* require urgent improvements in the monitoring and prevention of NDM-KP.

Compared to NDM-1, NDM-5 has greater hydrolytic activity toward carbapenems and expanded-spectrum cephalosporins (2, 4, 7, 8). Similarly, the transconjugant acquisition of *bla*_NDM-5_-carrying plasmid in our study displayed elevated cefepime, imipenem, and meropenem MICs compared with transconjugants with *bla*_NDM-1_-positive plasmid.

Clonal spread and plasmid horizontal transmission are the two common ways to spread carbapenemase-producing *K. pneumoniae*. The MLST and CgMLST results in our study revealed that NDM-KP isolates were not clonally related. The NDM-KP strains in this study have a surprising diversity of STs, and they are not clonally related. Many STs, such as ST11, ST14, ST15, and ST147, are common NDM-positive *K. pneumoniae* clonal lineages that have been found in multiple studies, but sufficient evidence to demonstrate that these STs are high-risk clones mediating the transmission of *bla*_NDM_ is lacking (9, 10).

*Bla*_NDM-1_ is mainly located on plasmids and plays a role in the dissemination and spread of resistance genes. *bla*_NDM-1_ and *bla*_NDM-5_ have been reported to be located on plasmids with many replicon types (11). The replicon types of *bla*_NDM-1_-carrying plasmids in *K. pneumoniae* included IncC, ColE10, IncFIA, IncFIB, IncHI1, IncHI3, IncN2, IncL/M, IncP, IncR, IncT, IncX1, IncX3, and IncY (2). For *bla*_NDM-5_-carrying plasmids, there is an equal variety of replicon types, such as IncFUA, IncFIB, Inc FIC, IncFII, IncX3, IncX4, and IncY. Of note, IncX3 appears to be the most popular replicon type of the *bla*_NDM_-carrying plasmid. To date, IncX3 plasmids carrying *bla*_NDM-1_ and *bla*_NDM-5_ have been reported worldwide. Most of the *bla*_NDM_-carrying IncX3 plasmids deposited in GenBank have been found in China and neighboring countries in East Asia (2).

The genetic environments of *bla*_NDM-1_ and *bla*_NDM-5_ in our study were nearly identical and were associated with △IS*Aba125* upstream and *ble*_MBL_ downstream. The similar genetic environment of *bla*_NDM-1_ and the backbones of the *bla*_NDM_-positive plasmid in this study suggest that *bla*_NDM-5_ may have evolved from *bla*_NDM-1_ because another copy of IS*26* was found in the *bla*_NDM-1_-harboring plasmid; one IS*26*-mediated deletion event might have happened, which contributed to the evolution from a *bla*_NDM-1_-harboring plasmid to a *bla*_NDM-5_-harboring plasmid (12). The IncX3 plasmid may be a major vehicle mediating the dissemination and evolution of *bla*_NDM_ variants.

IncX3 plasmids appear to be a potentially successful vehicle for spreading *bla*_NDM_. In Ma’s study, the majority of wild-type strains acquiring the IncX3 plasmid showed a low fitness cost in the absence of antibiotic selection pressure (13). Strains with plasmids carrying *bla*_NDM-1_ and *bla*_NDM-5_ in our study imposed significant but similar fitness costs on their hosts. We could not compare them with other plasmids and with other replicons due to the absence of other replicons in our study. Additionally, high transfer frequency was the other characteristic of the IncX3 plasmid. The conjugation frequency of the IncX3 *bla*_NDM_-carrying plasmid was between 10^−4^ and 10^−2^, which is consistent with the findings of Liu et al. (14). The transferability of the *bla*_NDM-1_-positive plasmid was greater than that of the *bla*_NDM-5_-positive plasmid. The basic structure of the *bla*_NDM-1_-harboring plasmid and the *bla*_NDM-5_-bearing plasmid is similar, and the core genes of the backbone including plasmid replication-related genes, conjugation-related genes, and conjugation/type IV secretion system (T4SS, with 11 genes, pilX1 to pilX11) were identical. In addition, differences between the two plasmids included chaperonin GroEL, class A extended-spectrum β-lactamase SHV-12, DeoR/GlpR transcriptional regulator, and NAD(P)-dependent oxidoreductase; however, these differential genes do not affect the transmissibility of plasmids. Furthermore, more ISs were identified in the *bla*_NDM-1_-bearing plasmid, such as more copies of IS*26*. This may be a cause of higher transmissibility.

### Conclusions

We described the molecular characterization of NDM-KP strains that belonged to various STs. Both *bla*_NDM-1_ and *bla*_NDM-5_ were located on a self-transmissible IncX3 plasmid, which could be transferred to *E. coli* with a low fitness cost and high transfer frequency. Moreover, the genetic environment of *bla*_NDM-1_ and the backbones of the *bla*_NDM_-positive plasmid was similar, suggesting that the IncX3 plasmid may be a major vehicle mediating the dissemination and evolution of *bla*_NDM_ variants.

## MATERIALS AND METHODS

### Bacterial strains

A total of 15 NDM-producing *Klebsiella pneumoniae* (NDM-KP) strains were collected from 1,376 CRKP isolates between 2019 and 2021 in two hospitals in Zhejiang, China. The MBL carbapenemase gene *bla*_NDM_ was screened by PCR using specific primers from the previous study and confirmed by Sanger sequence (15).

### Antimicrobial susceptibility testing

The MICs of antibiotics were determined by the broth microdilution method as per Clinical and Laboratory Standards Institute (CLSI) recommendations. The results of colistin and tigecycline were interpreted according to European Committee on Antimicrobial Susceptibility Testing (EUCAST) clinical breakpoints (www.eucast.org) and other antibiotics in Table 1 according to CLSI criteria (16). *E. coli* ATCC 25922 was used for the quality control strain.

### Whole-genome sequencing and analysis

These NDM-KP isolates were genome sequenced using HiSeqTM 2000 (Illumina Inc., San Diego, USA). Reads were *de novo* assembled into contigs using CLC Genomics Workbench (CLC Bio 8.0). Acquired resistance genes were analyzed by ResFinder database plasmid replicons PlasmidFinder (both at https://cge.cbs.dtu.dk/) and sequence typing (http://bigsdb.pasteur.fr/klebsiella/klebsiella.html).

### Long-read genome sequencing and analysis

Representative isolates of NDM-1 and NDM-5-producing KP, NB1827 and ZRY2400, were selected to sequence by long-read genome sequencing by a MinION Sequencer (Nanopore; Oxford, UK). The complete genome sequence was obtained by *de novo* hybrid assembly of both short (Illumina) and long reads using Unicycler v0.4.3 and Pilon v1.24. Short reads of other isolates were mapped to pZRY2400-NDM1 and pNB1827-NDM5 using CGView (17).

### Phylogenomic analysis of NDM-KP

The phylogenetic tree was based on the core genome which consisted of 4,143 genes defined by Panaroo (v.1.2.10) that existed in 99%–100% strains. The phylogenetic tree was constructed by RAxML-NG (v.1.0.1) based on the bootstrap analysis. We chose the autoMRE option, and the bootstrapping converged after 650 replicates (Fig. S1) (18). The virulence and antimicrobial resistance genes were annotated by Kleborate3 (v.2.2.0) with default options (19). The phylogenetic tree and gene heatmap were visualized by ggtree4 (20).

### Conjugation assay

Mating experiments were performed as previously described, and *E. coli* J53 was used as the recipient strain as described previously (15, 21, 22). Putative transconjugants were selected on MH agar plates supplemented with ampicillin (100  mg/L) and sodium azide (300  mg/L). The transconjugants were confirmed through PCR with specific primers (NDM_F58 5′-ggcggaatggctcatcacga-3′; NDM_R344 5′-cgcaacacagcctgactttc-3′).

### Growth kinetics

Growth curves for the recipients were performed in 96-well plates as described previously (23). The recipients J53/NDM-1 and J53/NDM-5 and J53 were diluted in LB broth medium in 96-well microtiter plates and incubated at 37°C for 24 h. OD600 measurements were taken hourly to construct a growth curve. Relative growth rates were measured via R script, and AUC values were calculated using GraphPad Prism software 8.0.2 (GraphPad Software, San Diego, CA, USA). Growth kinetics assays were performed in triplicate.

### Statistical analysis

Relative growth rates were evaluated using Student’s *t*-test. Statistical significance was analyzed using GraphPad Prism 8.0.2 (GraphPad Software, San Diego, CA, USA). *P* < 0.05 was considered statistically significant.

## Data Availability

The genome sequences used in this study have been deposited in the National Center for Biotechnology Information database under BioProject PRJNA936643.

## SUPPLEMENTAL MATERIAL

Fig. S1 spectrum.01028-23-s0001.tif The bootstrap analysis of core genome phylogenetic tree.

ASM does not own the copyrights to Supplemental Material that may be linked to, or accessed through, an article. The authors have granted ASM a non-exclusive, world-wide license to publish the Supplemental Material files. Please contact the corresponding author directly for reuse.
