# Supplementary figures and images for: Molecular characterization and comparison of bla NDM-1-carrying and bla NDM-5-harboring IncX3-type plasmids in carbapenem-resistant Klebsiella pneumoniae
Source: Microbiol Spectr. 2023 Aug 25;11(5):e01028-23. doi: 10.1128/spectrum.01028-23 (PMC10581223; doi:10.1128/spectrum.01028-23)

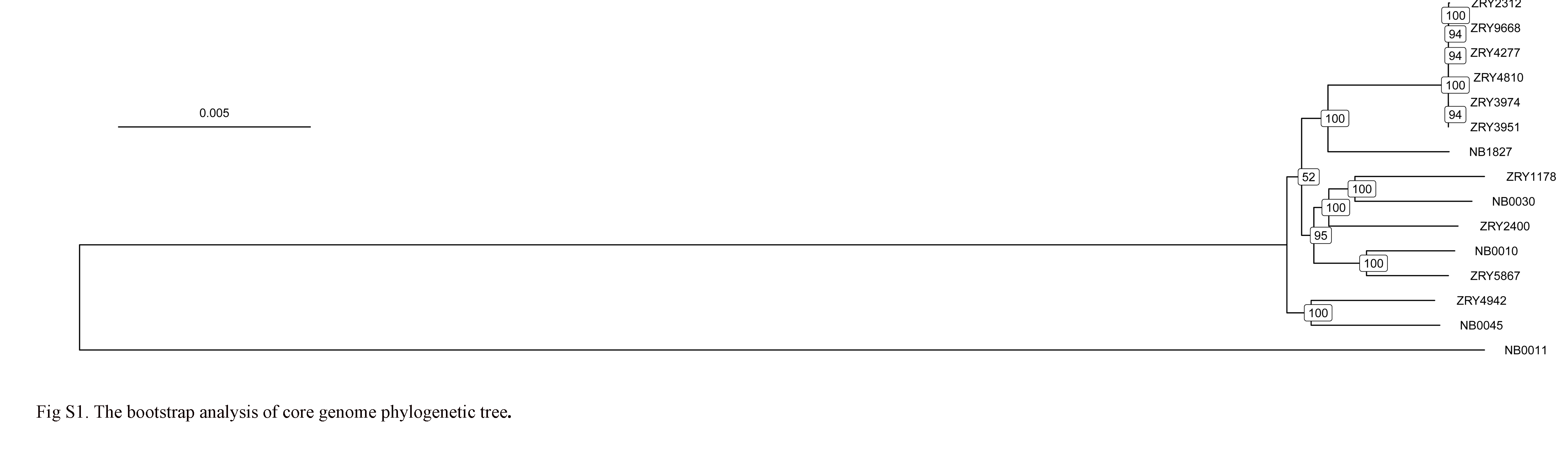

Supplement: Fig. S1 — The bootstrap analysis of core genome phylogenetic tree. [file spectrum.01028-23-s0001.tif]
